# Intraplaque neovascularization and progression of atherosclerosis: A review of SMI technical assessment and berberine-statin regulatory mechanisms

**DOI:** 10.1097/MD.0000000000046174

**Published:** 2025-11-21

**Authors:** Xv Sun, Fanbo Wang

**Affiliations:** a Jiamusi University, Jiamusi, Heilongjiang Province, China; b Department of Physical Diagnosis, The First Affiliated Hospital of Jiamusi University, Jiamusi City, Heilongjiang Province, China.

**Keywords:** atherosclerosis, berberine, ferroptosis, intraplaque neovascularization, SMI, statins

## Abstract

Intraplaque neovascularization (IPN) is a central driver of atherosclerosis (AS) progression and plaque vulnerability, and its dynamics are closely related to the risk of cardiovascular events. Superb microvascular imaging (SMI) has become a key tool for IPN assessment due to its noninvasive and highly sensitive characteristics, and the combination of berberine and statins has demonstrated significant synergistic effects in regulating IPN and stabilizing plaques. This article describes the pathological mechanisms of IPN in the progression of AS, summarizes the value of SMI technology and multimodal imaging in the assessment of IPN, and analyzes the molecular mechanisms and clinical evidence of berberine and statins in the regulation of IPN, so as to provide an integrated “assessment-intervention” basis for the precise prevention and treatment of AS.

## 1. Introduction

Atherosclerosis (AS) is the main pathological basis of cardiovascular and cerebrovascular diseases, and its core danger is acute thrombotic events triggered by plaque rupture, and the formation and development of intraplaque neovascularization (IPN) is a key driver of this process. As a new noninvasive ultrasound technique, superb microvascular imaging (SMI) can clearly show the low-velocity blood flow signal of IPN without contrast agent, which provides a breakthrough means to solve the limitations of traditional assessment techniques; and the combination of berberine and statins can synergistically regulate the formation of IPN through multi-targets, which provides a new mechanism to improve the stability of plaque. The combination of berberine and statins can synergistically regulate IPN formation through multi-targets, and its complementary mechanism provides a new strategy to enhance plaque stability. Based on the existing research, this paper focuses on the association between IPN and AS progression, the value of SMI technology in assessing pain points and the synergistic regulatory mechanism of the 2 classes of drugs, so as to provide theoretical support for the targeted treatment of AS. This review is based on the summary analysis of published literature and does not involve original clinical studies, so it does not need to be approved by the Ethics Committee and does not involve issues related to patients’ informed consent.

## 2. Mechanism of intraplaque neovascularization in the progression of atherosclerosis

IPN is an abnormally proliferating microvascular network within AS plaques, which mainly originates from the periplasmic trophoblastic vessels at the base of the plaque, and its formation is jointly regulated by hypoxia, inflammatory factors and angiogenic factors, which is a core pathological hallmark of AS progression.

### 2.1. Pathophysiologic mechanisms of IPN promoting AS progression

Zhao et al^[1]^ (2023) found that small and dense low-density lipoprotein cholesterol (sdLDL-C) was an independent risk factor for the formation of IPN (OR = 2.13, *P* < .01), and the IPN grade of SMI was positively correlated with the thickness of plaques (*R* = 0.62, *P* < .001), suggesting that IPN promotes the movement of lipid components such as sdLDL-C to plaques by increasing vascular permeability – a core pathophysiological hallmark of AS progression. This suggests that IPN increases vascular permeability, promotes the penetration of lipid components such as sdLDL-C into the plaque, accelerates the expansion of the lipid core, and then promotes the growth of plaque volume. Notably, sdLDL-C not only directly promotes IPN formation, but also upregulates IL-6 through activation of the NOD-like receptor thermoprotein structural domain-related protein 3 (NLRP3) inflammasome, forming a positive feedback loop of “lipid deposition-inflammation amplification-vascular neogenesis” (Zhao et al,^[1]^ 2023; Liu et al,^[2]^ 2020). Meanwhile, IPN provides a channel for the infiltration of inflammatory cells (e.g., macrophages, T cells), and the inflammatory factors, in turn, counteract IPN formation. Chen et al^[3]^ (2023) demonstrated that risk factors such as smoking and hypertension promoted IPN neogenesis by up-regulating the levels of tumor necrosis factor α (TNF-α) and interleukin-6 (IL-6) in the plaques and stimulating the expression of vascular endothelial growth factor, and the incidence of cerebral infarction in patients with SMI flow score ≥ 2 was 3.2 times higher than that of patients with a low score (*P* < .05), directly confirming that IPN is associated with inflammation, and that IPN formation is a major cause of inflammatory cell infiltration. The strong association between IPN and inflammation-driven plaque destabilization is directly confirmed. In conjunction with the study of Sun et al^[4]^ (2022), the serum neutrophil/lymphocyte ratio (NLR) was significantly elevated in IPN-positive patients, suggesting that inflammatory cell infiltration may exacerbate the risk of intraplaque hemorrhage. In addition, IPN has a weak vascular wall structure (lacking smooth muscle cells and intact basement membrane) and is prone to rupture and hemorrhage. Wang et al^[5]^ (2022) found in patients with acute cerebral infarction that the incidence of intraplaque hemorrhage was significantly higher in those with positive IPN (42.3%) than in those with negative IPN (11.5%) and the consistency between SMI and contrast-enhanced ultrasound (CEUS) for diagnosis of IPN was high (Kappa = 0.723 for ACI group), further suggesting that IPN is an important predictor of intraplaque hemorrhage. Ultrasound (CEUS) to diagnose IPN (Kappa = 0.723 in ACI group), further suggesting that IPN is an important predictor of intraplaque hemorrhage.

### 2.2. IPN as a marker of AS progression staging

From the perspective of AS progression staging, the density and distribution of IPN are characteristic: IPN in early plaques (lipid pattern stage) is rare, IPN in progressive plaques (fibrous plaque stage) begins to extend from the outer membrane to the plaque core, and IPN in vulnerable plaques (complex plaque stage) is dense and mostly distributed around the lipid core. Zhao^[6]^ (2024) et al observed by SMI that the IPN generation rate of mixed-echo plaques (78.6%) was significantly higher than that of isoechoic plaques (23.1%), and that the risk of cerebral infarction increased 1.8-fold for every 1-point increase in the IPN score (*P* < .01), which confirms that the IPN can be used as a reliable imaging marker in the progression staging of AS. It is worth noting that the association between IPN and systemic metabolism and complications has become increasingly clear, suggesting that IPN can be used as an early warning indicator of systemic vascular complications, and the concordance between its AP score and SMI for detecting IPN was as high as Kappa = 0.806, which provides a multidimensional validation of the clinical application of IPN. To further visualize the core evidence of IPN in clinical association and drug efficacy assessment, the key data of related studies are summarized below (Table 1).^[7,8]^ The following studies confirm that IPN is not only an early warning indicator of vascular complications, but also a reliable marker for drug efficacy assessment.

**Table 1:** Summary of core data from IPN-related clinical studies.

Research purpose	Key indicators	Core results (HR/OR value or efficacy difference)	Sample size/follow-up time	Literature source
Association of IPN with diabetic complications	Risk of microvascular lesions	OR = 3.68 (95% CI: 2.11–6.42)	236 T2DM patients	Cheng et al (2022)^[7]^
IPN combined with NLR for predicting cerebral infarction	Predictive sensitivity/specificity	87.5%/77.3% (combined detection)	156 cerebral infarction patients	Sun et al (2022)^[4]^
Combined efficacy of berberine and statins	Crouse score (sum of thicknesses of each independent plaque in bilateral carotid arteries)	The combined group was significantly lower than the single-drug group (*t* = 4.186, *P* < .01)	90 patients	Liu (2020)^[8]^

CI = confidence interval, Crouse integral = the sum of the thickness of each individual plaque in both carotid arteries, IPN = intraplaque neovascularization, NLR = neutrophil/lymphocyte ratio, OR = ratio of ratios, T2DM = type 2 diabetes mellitus.

## 3. The value of SMI technology in the assessment of intraplaque neovascularization

SMI technology separates low-velocity blood flow signals through advanced clutter suppression algorithms to achieve high-resolution visualization of IPN, and its value in IPN detection, grading, and clinical prognosis prediction has been verified by several studies, and its combined application with multimodal imaging can further enhance the comprehensiveness of the assessment.

### 3.1. Diagnostic efficacy of SMI (compared with other techniques)

SMI demonstrates significant advantages over the invasive and safety limitations of traditional assessment techniques. In terms of diagnostic efficacy, Guo et al^[9]^ (2024) used surgical pathology as the gold standard and found that the sensitivity of SMI in diagnosing IPN was 93.83%, the specificity was 89.47%, the Kappa value was 0.78, and there was a significant positive correlation between the SMI score and the pathologic microvessel density (MVD) (*R* = 0.76, *P* < .001). A study by Yan et al^[10]^ (2023) further confirmed that the concordance between SMI and pathologic diagnosis (Kappa = 0.875) and the area under the ROC curve (0.787) suggested that it could be used as a reliable diagnostic tool for IPN. Compared with traditional Contrast-Enhanced Ultrasound (CEUS), SMI is not dependent on contrast agents, avoids the risk of allergy and nephrotoxicity, and is especially suitable for people with contraindications to contrast agents (e.g., patients with renal insufficiency), and the 2 have comparable efficacies in the assessment of IPN. Sun et al^[11]^ (2025) demonstrated that SMI was significantly better than conventional ultrasound in the assessment of carotid stenosis and plaque properties using DSA as the gold standard. The core principles, advantages, and scenarios of different ultrasound technologies in IPN assessment are significantly different, as shown in Table 2.^[12–16]^

**Table 2:** Comparison of the characteristics of different ultrasound techniques for the assessment of intraplaque neovascularization (IPN).

Technology type	Core principle	IPN assessment advantages	Limitations	Applicable scenarios	Literature support
SMI	Suppress clutter signals and capture low-velocity blood flow	Noninvasive, no contrast agent required, and long-term follow-up possible	Signal attenuation in obese patients (sensitivity 78.2% when BMI > 30)	Screening of asymptomatic populations, and for those with contrast agent contraindications	Guo et al. (2024),^[9]^ Cheng et al (2023)^12]^
CEUS	Enhance blood flow signals with contrast agent	High sensitivity (71.2%) and good consistency with pathology	Requires contrast agent and has allergy risk	Diagnosis of vulnerable plaques and short-term efficacy assessment	Chen et al (2023),^[3]^ Jiang et al. (2022)^[3]^
SWE	Assess tissue stiffness (Young modulus)	Can simultaneously determine fibrous cap thickness/ lipid core hardness	Cannot directly display IPN blood flow	Comprehensive assessment of suspected vulnerable plaques (combined with SMI)	Zamani et al (2020),^[14]^ Zeng et al (2024)^[15]^
Conventional ultrasound	Show plaque morphology with gray-scale imaging	Simple operation and low cost	Poor sensitivity to low-velocity blood flow (IPN detection rate 39%)	Preliminary screening of plaques	Forsberg et al (2019)^[16]^

BMI = body mass index, CEUS = contrast-enhanced ultrasound, DSA = digital subtraction angiography, IPN = intraplaque neovascularization, MVD = microvessel density, SMI = superb microvascular imaging, SWE = shear-wave elastography.

### 3.2. Multimodal imaging combined assessment strategy

To compensate for the limitations of a single technique, the combination of SMI and multimodal imaging has become a key strategy to solve the problem of accurate IPN assessment. Cheng et al^[12]^ (2023) pointed out that B-mode ultrasound can be used as a basic imaging to show the plaque morphology, whereas SMI and CEUS can focus on the detection of IPNs, and shear-wave elastography (SWE) can assess plaque stiffness by Young modulus. A prospective multimodal study by Zamani et al^[14]^ (2020) further demonstrated that SMI in combination with MRI and PET can improve the accuracy of carotid plaque instability assessment, suggesting that the combined “neovascularization-tissue stiffness” metrics can optimize the identification of vulnerable plaques.

From the perspective of clinical practice, multimodal combination strategies in different scenarios have their own advantages: SMI combined with B-mode ultrasound can be used as a primary screening tool for asymptomatic high-risk populations (e.g., patients with hypertension or diabetes)^[12]^; in contrast, for patients with suspected vulnerable plaques (e.g., those with a history of transient ischemic attack), a combined SMI + SWE assessment is recommended,^[14]^ in which SMI quantifies IPN density and SWE determines fibrous cap stiffness, and the combination of the 2 results in improved stroke risk prediction (Zeng et al,^[15]^ 2024).

### 3.3. Clinical prognostic value and operation procedure recommendations

SMI can effectively predict plaque vulnerability and prognosis. In a follow-up of 96 patients with carotid plaques, Ma et al^[17]^ (2022) found that the density of IPN detected by SMI was positively correlated with the incidence of ischemic stroke (OR = 4.12, *P* < .001), and that those with a plaque thickness of >4 mm and an SMI score of ≥2 had an increased risk of stroke by 6.3 times. A study by Fan^[18]^ (2024) also showed that the IPN grade was significantly higher in the symptomatic group than in the asymptomatic group (mean 2.1 vs 0.8, *P* < .001), and that a high IPN grade was an independent risk factor for the severity of AS (OR = 3.17, *P* < .001).

In addition, SMI can reflect the effect of interventions on IPN in real time. Qi et al^[19]^ (2022) found high concordance between SMI scores and CEUS scores (Kappa = 0.76) and the magnitude of score decrease was synchronized with the improvement of plaque stability after 6 months in atorvastatin treatment; Zeng et al^[20]^ (2021) further demonstrated that SMI can be a sensitive measure of the severity of IPN after atorvastatin treatment. Zeng et al^[20]^ (2021) further demonstrated that SMI can sensitively capture the reduction of IPN number (mean reduction of 2.3/plaque) and the weakening of blood flow signals after atorvastatin treatment, which provides a quantitative basis for the evaluation of efficacy.

Based on the above evidence, the following clinical procedure is recommended:

①. For people at high risk of AS (e.g., those with hypertension, diabetes mellitus, and a history of smoking), the first screening should use SMI combined with B-mode ultrasound to clarify the IPN classification;
②. If the SMI score is < 2 (low risk), repeat the examination once a year to monitor the IPN dynamically;
③. If the SMI score is ≥ 2 (high risk), the examination should be repeated every 6 months, and the fiber cap stiffness should be evaluated with SWE to predict the risk of stroke;
④. During pharmacologic interventions (e.g., berberine combined with statin), IPN improvement is assessed by SMI every 3 to 6 months until the score falls below 1, which can be extended to annual review.

In their review, Zeng et al^[15]^ (2024) emphasized that the semi-quantitative/quantitative grading of IPN by SMI can be directly used to predict stroke and assess drug efficacy, but the problem of inconsistent quantification standards needs to be solved, and the integration of artificial intelligence technology is expected to achieve automatic analysis of IPN vascular density and branching complexity, and to solve the problem of standardization.

## 4. Mechanisms and clinical evidence of berberine and statins in regulating neovascularization in plaques

The formation of IPN is regulated by lipid metabolism, inflammatory pathways and angiogenic factors, etc. Berberine and statins can inhibit IPN and stabilize plaques by targeting these links, and the combination of berberine and statins has a synergistic effect.

### 4.1. Individual action of statins

In response to the lipid-driven mechanism of IPN formation, statins inhibit IPN through both lipid-lowering and vasoprotective effects. Li et al^[21]^ (2024) compared 20mg/d with 40mg/d of atorvastatin and found that after 3 months of treatment in the high-dose group, there was a significant reduction in the low-density lipoprotein (LDL) cholesterol (LDL-C) (38.6% vs 25.3%, *P* < .05) and the IPN grade (1.2 vs 0.7, *P* < .05). The decrease in IPN grade (grade 1.2 vs grade 0.7, *P* < .05) was more significant, suggesting that the lipid-lowering effect could reduce the lipid drivers of IPN. The core mechanism is to inhibit the internalization of angiopoietin 2 and VE-calmodulin, which improves vascular maturation (increased pericyte coverage) and reduces the promotion of IPN formation by LDL-C.

### 4.2. Berberine alone

In response to the ferroptosis mechanism of IPN vascular endothelial cells, berberine blocks IPN progression through specific targeting. Hong et al^[22]^ (2024) demonstrated in an ApoE−/− mouse model that berberine specifically inhibits the activity of lipoyl coenzyme A synthetase 4 (ACSL4) and reduces the lipid peroxidation of polyunsaturated fatty acids such as arachidonic acid. Unsaturated fatty acid lipid peroxidation, thus blocking endothelial cell ferroptosis; at the same time, berberine up-regulated glutathione peroxidase 4 (GPx4) expression and maintained cellular redox balance. This study further revealed that endothelium-specific overexpression of ACSL4 completely reversed the protective effect of berberine on the endothelium of IPN vessels, suggesting that ACSL4 is a key target of berberine in regulating the stability of IPN vessels. In addition, berberine can promote cholesterol efflux through activation of PPARγ and further inhibit IPN formation.

### 4.3. Synergistic mechanism

The 2 classes of drugs form a synergistic network through complementary targets, which solves the problem of the limited effect of a single drug in regulating IPN. Clinical studies have demonstrated that the combination of the 2 classes of drugs can enhance IPN inhibition through a complementary mechanism. The 2 drugs form a synergistic network through complementary targets, which solves the problem of the limited effect of a single drug in regulating IPN. The specific synergistic mechanisms are shown in Table 3.

**Table 3:** Mechanisms and synergistic effects of berberine and statins on IPN regulation.

Drug type	Core target/pathway	Regulatory effect on IPN	Clinical evidence (efficacy indicator)	Synergistic target/complementary effect
Statins (e.g., atorvastatin)	Inhibit angiopoietin 2 and VE-cadherin internalization	Improve vascular maturity (increase in pericyte coverage) and reduce LDL-C	IPN grade decreased by 1.2 levels (40mg/d, Li et al,^[21]^ 2024)	Cooperate with berberine to inhibit the NF-κB pathway and enhance anti-inflammatory effects
Berberine	Inhibit ACSL4 and activate PPARγ	Block endothelial ferroptosis and promote cholesterol efflux	Combined with statins, the reduction amplitude of plaque thickness increased by 40% (Liu,^[8]^ 2020)	Reverse the decrease in statin sensitivity induced by high glucose (Zhao,^[23]^ 2020)

ACSL4 = adipoyl coenzyme A synthase 4, IPN = intraplaque neovascularization, LDL-C = low-density lipoprotein cholesterol, MAPKs = mitogen-activated protein kinases, NF-κB = nuclear factor κB, PPARγ = peroxisome proliferator-activated receptor γ, TC = total cholesterol, TG = triglycerides.

Lan et al^[24]^ (2018) divided patients with hypertension combined with AS into a control group (atorvastatin) and a berberine combination group, and found that after 8 weeks, the reduction of TC, TG, and LDL-C in the combination group was significantly higher than that in the control group (*P* < .05), and the effect of high-dose berberine (0.6 g/ times, 3 times/d) was better, suggesting that the combination of drugs improves the lipid disorders and strengthens the inhibition of the This suggests that the combination of drugs can improve lipid disorders through multiple pathways and strengthen the inhibition of IPN lipid drivers. Chen et al^[25]^ (2014) further demonstrated that the LDL cholesterol compliance rate and Crouse score decreased more in the combination group, which could enhance plaque stability.

Meanwhile, Zhao^[23]^ (2020) found a higher overall effective rate (92.59% vs 81.48%, *P* < .05) of atorvastatin combined with berberine in patients with diabetes mellitus combined with cerebral infarction. This synergistic effect is closely related to the combined inhibition of inflammatory pathways: high glucose-induced oxidative stress can activate the nuclear factor κB (NF-κB) pathway, which can weaken the inhibitory effect of statins on inflammatory factors, while berberine inhibits mitogen-activated protein kinases (MAPKs) through the inhibition of the MAPKs, which are the most effective inhibitors of inflammation in cerebral infarction. kinases (MAPKs) and NF-κB pathway activation (Yang et al,^[26]^ 2023), which can reverse this process and form a synergistic inhibition of the NF-κB pathway with statins, thus maintaining the efficacy of IPN regulation under hyperglycemia (Li et al,^[27]^ 2016), and has a good safety profile without serious adverse effects.

### 4.4. Limitations of existing clinical evidence and preliminary references

It should be noted that the existing clinical evidence still has the limitation of a short follow-up period: the study by Lan et al^[24]^ (2018) had a follow-up period of only 8 weeks, which only reflected the acute inhibitory effect of the combination on lipid metabolism and IPN grading, and was unable to adequately validate the maintenance of plaque stability by long-term use of the drug (e.g., whether it continued to block the vascular endothelial ferroptosis pathway, whether it reduced the risk of hard endpoint events such as ischemic stroke or myocardial infarction for more than 1 year), and it was also difficult to rule out possible dose-related adverse effects (e.g., liver function abnormalities, creatine kinase elevation, etc) of long-term use. or myocardial infarction, and it is difficult to rule out possible dose-related adverse effects of long-term use (e.g., abnormalities in liver function, elevation of creatine kinase), resulting in limited clinical extrapolation of conclusions.

Despite the lack of long-term large-sample data, small-sample studies provide an initial reference for efficacy stability: small-sample follow-up by Liu^[8]^ (2020) and Zhao^[23]^ (2020) showed continuity of short-term efficacy of the combination and good tolerance in complex populations such as diabetes mellitus, which indirectly corroborates the long-term tolerability of the drug in complex metabolic states such as hyperglycemia. These small-sample data support the hypothesis of “long-term benefits of drug combinations,” but need to be further validated in large-sample long-term studies.

### 4.5. Recommendations for medication selection based on population characteristics

①. Patients with diabetes mellitus combined with AS: Prefer berberine (0.6g/d in 3 doses) combined with medium-dose statin (atorvastatin 20mg/d). Zhao^[23]^ (2020) demonstrated that this regimen could reverse high glucose-induced NF-κB pathway activation and improve statin resistance, with an overall effective rate of 92.59%, which was significantly higher than that of the single-agent group (81.48%);
②. Hypertension combined with AS patients: berberine (0.6g/ times, 3 times/d) combined with atorvastatin (20–40mg/d) is recommended. Lan et al^[24]^ (2018) found that the combination could reduce TC, TG, and LDL-C by multiple pathways, and the improvement of dyslipidemia was more significant with high doses of berberine;
③. Ordinary AS patients (without underlying disease): berberine (0.3–0.6g/d) can be used in combination with low- and medium-dose statin (atorvastatin 10–20mg/d).

## 5. Challenges and future directions for current research

### 5.1. Core challenges of current research

In terms of SMI technology, Zhao et al^[28]^ (2024) pointed out that the grading of IPN by SMI is mostly semi-quantitative (grades 0–3), and lacks uniform quantitative standards (e.g., vascular density, blood flow velocity), and the results of different centers are poorly comparable; Hou et al^[29]^ (2024) also mentioned that the differences in operators’ experience and equipment parameters may lead to bias in the identification of IPN, and that standardized operation procedures need to be established. Zeng et al^[15]^ (2024) further pointed out that there is a lack of a unified model for parameter integration in multimodal imaging (e.g., SMI combined with SWE).

In terms of drug regulation mechanisms, most existing studies focus on lipid metabolism and inflammatory pathways, while the effects of berberine and statins on IPN vascular endothelial cell function and pericyte recruitment have not yet been clarified; and the optimal dosage ratio and long-term effects of the combination of drugs are not supported by large-sample studies, e.g., the follow-up of the study of Lan et al^[24]^ (2018) was only 8 weeks, which is difficult to reflect the long-term efficacy. In addition, the association between IPN and systemic metabolism has been insufficiently studied. Zhao et al^[1]^ (2023) found that the TG/HDL-C ratio was associated with IPN, but the association between IPN and insulin resistance and intestinal flora metabolites (e.g., TMAO) has not yet been verified by SMI technology, which limits the understanding of the multi-systemic regulation of AS.

### 5.2. Ideas for the construction of multimodal image integration model

To address the lack of a unified model for multimodal imaging parameter integration, combining the characteristics of existing technologies and clinical needs, a “structure-function-metabolism” 3-dimensional integration model can be constructed, with a specific framework from the core logic, parameter design, and validation of the path in 3 aspects:

#### 5.2.1. Core logic of 3D integration modeling

SMI focuses on IPN vascular density (structural dimension), and Guo et al^[9]^ (2024) demonstrated that it is highly correlated with pathological microvascular density (MVD) (*R* = 0.76, *P* < .001), which can accurately reflect the status of plaque blood supply; SWE quantifies the tissue stiffness (functional dimension), and Zeng et al^[15]^ (2024) pointed out that SWE quantifies tissue stiffness (functional dimension) via Young modulus, which is directly related to fibrous cap stability – low stiffness indicates a thin, brittle cap and correlates with plaque rupture risk, and low stiffness suggests that the cap is thin and brittle, which is associated with the risk of plaque rupture; and MRI captures the metabolic characteristics of the lipid core (metabolic dimension). The combination of these 3 modalities can make up for the limitations of a single modality (e.g., SMI is unable to assess the overall plaque stiffness and SWE is unable to directly show the distribution of blood vessels), and realize a comprehensive assessment of the “microvascular structure to plaque function to metabolic status.”

#### 5.2.2. Scenario-based scenarios for parameter weight assignment

Scenario 1: Predicting plaque bleeding risk (core requirement: to identify IPN-related bleeding potential): focus on the inclusion of SMI vascular density (Wang et al,^[5]^ 2022, demonstrated that IPN positivity is significantly associated with the risk of plaque bleeding), SWE stiffness (low stiffness suggests that the vessel wall is fragile and susceptible to rupture), and MRI lipid core features (an enlarged lipid core may indirectly increase the risk of IPN stress), with weights assigned according to the “association of bleeding risk.” The weights were assigned according to the “bleeding risk association”.

Scenario 2: Evaluate the effect of drug intervention (core requirement: to judge the improvement of overall plaque stability): focus on the rate of change of SMI vessel density (reflecting the degree of inhibition of IPN by drugs), the rate of change of SWE stiffness (reflecting the effect of fibrous cap repair) and the rate of shrinkage of the lipid core of MRI (reflecting the improvement of lipid metabolism), and assign the weight according to the “response to the effect of the sensitivity of treatment.” “ side allocation.”

#### 5.2.3. Critical path of model validation

The reliability of the model should be ensured through the 2-dimensional verification of “pathology-clinical”:

① Pathological validation: take the pathological results of surgically resected plaques as the gold standard (e.g., SMI vascular density corresponds to pathological MVD, SWE hardness corresponds to collagen content), validate the correlation between the model output values and pathological indexes, and refer to the idea of the pathological control study of Guo et al^[9]^ (2024), to ensure that the results of the model assessment are consistent with the nature of the pathology;
② Clinical prognosis validation: track cardiovascular and cerebrovascular events (e.g., ischemic stroke, myocardial infarction), analyze the correlation between model scores and event rates, and clarify the predictive value of the model for clinical prognosis;

The validation process can refer to the multimodal research method of Cheng et al^[12]^ (2023), in which the details of the model are optimized through single-center preliminary validation, and then multicenter collaboration is carried out (covering different device brands and population characteristics) to promote the standardization of the model.

### 5.3. Solutions for SMI technology in IPN hierarchical standardization

To address the core problem of SMI technology in IPN grading standardization, combining with the existing research, the solution can be promoted from the 3 aspects – “clarifying the limitations, landing path, and verifying the direction”:

First, clearly define the correlation between the limitations of semi-quantitative grading and quantitative requirements. Currently, IPN grading in SMI is mainly semi-quantitative (grades 0–3), and lacks quantitative indexes such as vessel density and blood flow velocity (Zhao et al,^[28]^ 2024), which affects the comparability of assessment results between different centers (Hou et al,^[29]^ 2024); especially in cross-center studies, the consistency of grading has not yet reached the ideal level in clinical practice, and it is difficult to form a unified conclusion, which highlights the necessity of establishing a quantitative standard. This highlights the necessity of establishing quantitative standards.

Second, the feasibility of establishing quantitative standards should be improved through the dual path of “technical standardization + operational standardization.” On the technical level, the deep learning algorithm proposed by Zeng et al^[15]^ (2024) can be used to train an automatic quantification model based on multicenter carotid plaque SMI images to achieve objective calculation of IPN vessel density, branch complexity, and other indexes, and to reduce the bias of subjective judgments (this algorithm has been proven to improve the consistency of SMI image analysis); (on the operational level, reference should be made to the recommendations of Hou et al^[29]^ 2024), we formulated a unified SMI scanning standard operation procedure, clarified the key control parameters (e.g., probe frequency, blood flow gain) and positional specifications, and required the equipment to be calibrated periodically to reduce the interference of the equipment parameters and operating habits on the low-speed blood flow signal.

Finally, the clinical value of establishing quantitative criteria was verified through multidimensional validation. A multicenter collaborative study can be conducted to verify the correlation between AI-quantified IPN vascular density and pathological MVD, using microvascular density (MVD) detected by surgical pathology as the gold standard (Guo et al,^[9]^ 2024); at the same time, tracking cardiovascular and cerebral vascular events and exploring the correlation between the “quantitative threshold for IPN” and clinical prognosis, with the ultimate goal of forming an industry-recognized SI quantitative standard. The ultimate goal is to form an industry-recognized SMI-IPN quantitative grading standard to provide a unified basis for cross-center research and clinical decision-making.

Future research can be promoted in 3 aspects: first, to promote the precision and intelligence of SMI technology, combined with artificial intelligence technology to achieve automatic quantification of IPN, Zeng et al^[15]^ (2024) proposed that deep learning algorithms can improve the consistency of SMI image analysis (accuracy of 92%), providing a standardized tool for dynamic monitoring of the IPN; and at the same time, it is necessary to establish a fusion model of multimodal images (SMI + SWE + MRI) to achieve a comprehensive assessment of “structure-function-metabolism.” At the same time, it is necessary to establish a multimodal image (SMI + SWE + MRI) fusion model to realize the all-round assessment of “structure-function-metabolism.” Secondly, the key targets of berberine-statin regulation of IPN (e.g., NLRP3, ACSL4) should be explored through transcriptomic and metabolomic technologies, the molecular network of synergism should be clarified, and long-term follow-up studies (≥1 year) should be carried out to validate the effects of the combination on IPN and cardiovascular and cerebrovascular events. Thirdly, we will combine IPN characteristics detected by SMI with traditional risk factors (e.g., hypertension, diabetes) and biomarkers (e.g., sdLDL-C, hs-CRP, NLR) to establish a risk prediction model for AS progression, which will provide a basis for individualized intervention.

## 6. Conclusion

As an “assessable and interventable” target for the precise prevention and treatment of AS, the clinical management of IPN relies on the standardized assessment of SMI technology and the synergistic regulation of berberine and statin, which effectively solves the invasiveness and safety problems of traditional IPN assessment through its noninvasive and highly sensitive characteristics, and provides a reliable means of dynamic monitoring of IPN by combining with multimodal imaging. SMI technology effectively solves the problems of invasiveness and safety of traditional IPN assessment through its noninvasive and highly sensitive characteristics, and provides a reliable means for dynamic monitoring of IPN by combining with multimodal imaging; berberine (especially targeting ACSL4 to inhibit the endothelial ferroptosis) and statins, on the other hand, have cracked the difficult problem of the limited effect of a single drug in IPN regulation through synergistic improvement of lipid metabolism, inhibition of inflammation and angiogenesis, which has demonstrated a significant advantage in stabilizing plaques. Forming a closed loop of “assessment-intervention-monitoring”: for vulnerable plaque patients with dense IPN (score ≥ 2) detected by SMI, berberine (0.6g/d) combined with atorvastatin (20mg/d) is recommended, and IPN changes are monitored by SMI every 3 months until the score falls below 1, in order to reduce the risk of stroke; for patients with low risk (score < 2), they will be reviewed by SMI annually, so as to realize the whole process of precise management of AS. For low risk patients (score < 1), the SMI is reviewed annually to realize the accurate management of AS.

## Author contributions

**Resources:** Xv Sun.

**Software:** Xv Sun.

**Supervision:** Xv Sun, Fanbo Wang.

**Validation:** Xv Sun.

**Visualization:** Xv Sun.

**Writing – original draft:** Xv Sun.

**Writing – review & editing:** Xv Sun.
